# Editorial: Organ transplantation by 2040

**DOI:** 10.3389/frtra.2026.1861786

**Published:** 2026-05-25

**Authors:** Jerzy W. Kupiec-Weglinski, Ronald W. Busuttil

**Affiliations:** The Dumont-UCLA Transplantation Center, Department of Surgery, Division of Liver and Pancreas Transplantation, David Geffen School of Medicine, UCLA, Los Angeles, CA, United States

**Keywords:** organ transplantation, organ shortage, organ preservation, transplant immunology, transplant policy

Advances in organ transplantation continue to redefine the boundaries of modern medicine (1). Yet persistent challenges, from organ shortages to immune-mediated graft injury, underscore the need for innovation across clinical, biological, and societal domains. Framed by the forward-looking theme of “*Transplantation by 2040*”, the body of work published in this Frontiers Research Topic reflects a field undergoing rapid conceptual and technological transformation worldwide. This transformation is driven by mechanistic insights, data integration, and a shift toward precision medicine.

A fundamental limitation remains the insufficient supply of donor organs. A cross-sectional study of healthcare professionals in Mexico by the Monterrey group of Dr. Reyna-Sepulveda highlights a critical gap between favorable attitudes toward donation and inadequate knowledge of the donation process. Despite a high willingness to donate, substantial proportions of respondents lacked awareness of key procedural elements, including the role of family consent, and expressed concerns about donor morbidity. Importantly, strong support for socioeconomic protection of living donors suggests that structural interventions may enhance donation rates without undermining altruistic motivation.

Building on this, Dr. Zeybek's multinational team's analysis of societal and policy contexts illustrates how political affiliation and attitudes in the German Bundestag influence organ donation policy, particularly the choice between opt-in and opt-out systems. The observed heterogeneity in both opinion expression and voting behavior suggests that legislative outcomes may not consistently reflect an evidence-based consensus, reinforcing the need for sustained engagement between policymakers and the medical community. These findings emphasize that expanding the donor pool will require not only biomedical innovation but also evidence-based policy, targeted education, and public trust-building initiatives.

At the clinical frontier, transplantation is increasingly able to address highly complex and previously prohibitive conditions. For example, Dr. Vivek Vij's Indian team reported a case of ABO-incompatible living donor liver transplantation in a pediatric patient with Rosai–Dorfman–Destombes disease, exemplifying the expanding scope of transplant feasibility. The successful use of desensitization strategies, including B-cell depletion and immunoadsorption, reflects the growing capacity to overcome immunological barriers through individualized protocols. These cases highlight the clinical evolution and foreshadow a future in which transplantation is increasingly tailored to patient-specific immunological and pathological contexts.

The Pittsburgh group of Dr. Thomson discusses how a refined understanding of alloimmunity is reshaping the future of transplantation by moving beyond conventional paradigms of broad immunosuppression. While traditional approaches have primarily targeted adaptive immune responses, growing evidence highlights the central role of innate immune cells in regulating graft injury and tolerance. High-resolution technologies, including single-cell RNA sequencing and spatial transcriptomics, have revealed substantial heterogeneity within these populations, enabling the identification of regulatory subsets with tolerogenic potential. These insights are driving the development of innovative therapies aimed at selective immune reprogramming, such as modulation of myeloid inhibitory checkpoints, metabolic reprogramming, and adoptive transfer of regulatory immune cells. Together, these strategies hold promise for achieving durable graft tolerance while reducing dependence on lifelong immunosuppression.

Building on recent immunological insights, mechanistic connections have become clearer. These immunological processes are closely linked to regulated cell death (RCD) and the release of damage-associated molecular patterns (DAMPs), which serve as key initiators of innate immune activation. The reviewed framework, which integrates RCD and DAMP biology, provides a unifying model linking donor ischemia–reperfusion injury and early allograft dysfunction, as discussed by the Munich-based Professor Land and the Heidelberg-based Professor Linkermann. Multiple forms of RCD, including ferroptosis, necroptosis, and pyroptosis, generate DAMPs that activate pattern recognition receptors and amplify inflammatory signaling cascades. Looking ahead, these pathways are particularly significant as both biomarkers and therapeutic targets, enabling real-time assessment of organ quality and the development of targeted interventions to mitigate injury before implantation.

Two additional reports examine the limitations imposed by poor donor organ quality. The Oxford team, led by Professor Peter Friend, provides an update on how organ preservation technologies are advancing rapidly alongside progress in immunology and mechanistic research. Indeed, machine perfusion has already enabled a shift from static preservation to dynamic, *ex vivo* organ optimization. Normothermic perfusion, real-time biomarker assessment, and artificial intelligence could enable precise evaluation of organ viability and targeted regenerative therapies before implantation. Centralized assessment and reconditioning centers will integrate logistics, diagnostics, and therapeutics into a unified transplantation ecosystem.

Research on donor-specific graft injury, as discussed by Dr. Tebbutt's group in Vancouver, Canada, indicates that interventions should be tailored to donor type, distinguishing between donation after brain death and donation after circulatory death. Such stratification helps maximize the use of marginal organs and improve transplant outcomes. *Ex vivo* graft manipulation, including gene editing, targeted drug delivery, and cell engineering, may routinely convert discarded organs into transplantable grafts, substantially expanding the donor pool. However, both contributions emphasize that the full clinical potential of *ex vivo* graft manipulation will depend on rigorous trials and a deeper mechanistic understanding of peri-transplant organ injury. Integrating donor-specific molecular profiling with organ-rejuvenating adjunctive anti-inflammatory and cytoprotective therapies should enable more effective organ recovery. Collectively, these advances offer a path to expanding the donor pool, improving graft performance, and achieving better long-term outcomes.

Alongside technological and immunological advances, clinical outcomes in special populations remain critical. A meta-analysis of pregnancy after kidney transplantation, conducted jointly by the British and Greek teams of Dr. Vassilios Papalois, shows increased risks of maternal and neonatal complications, including higher rates of preeclampsia, preterm birth, and fetal mortality. A comprehensive review by Dr. Stelmach of Warsaw Medical University, in collaboration with UCLA, demonstrates that women after liver transplantation consistently achieve successful pregnancies and deliver healthy infants, but require precise multidisciplinary care. Both studies highlight transplantation's role in restoring quality of life and the ongoing need for specialized care and long-term monitoring for complex patients. Nevertheless, this field remains underexplored. Key questions include which birth control methods, especially hormonal ones, are safest and most effective; why pregnancy complications are more common in transplant recipients and how to prevent them; and how immunosuppressive therapies affect infants' long-term health, particularly in relation to breastfeeding recommendations.

Across policy, biology, technology, and clinical care, a common theme emerges: transplantation is no longer a single surgical intervention but a continuum of care spanning donor selection, organ preservation, immune modulation, and long-term patient management. Integrating novel biological insights, including the roles of innate immunity, metabolic signaling, and cell death pathways, with technological advances in organ perfusion and clinical monitoring is driving the field toward greater precision and personalization.

Despite this progress, the studies reviewed here highlight persistent gaps. Translating preclinical findings into clinical practice remains limited, particularly for targeted immunotherapies and biomarker-driven decision-making. Differences in patient populations, donor characteristics, and healthcare systems complicate the standardization of care. In addition, societal and political factors influence organ donation, underscoring the need for interdisciplinary approaches that extend well beyond biomedical innovation.

The future of transplantation depends on integrating precision immunology, innovative organ preservation techniques, and informed policy frameworks. A deeper understanding of the molecular and cellular mechanisms of graft injury and tolerance, together with innovations in donor utilization and patient-specific care, is essential to improving long-term outcomes. As the field advances, interdisciplinary collaboration, robust clinical investigation, and alignment among scientists, the biotech industry, and policymakers are essential to realizing the full life-saving potential and success of organ transplantation.

## References

[1] Kupiec-Weglinski JW. Grand challenges in organ transplantation. Front Transplant. (2022) 1:897679. 10.3389/frtra.2022.897679. eCollection 2022. 38994397 PMC11235338

